# Trusting the Voice? When an Attractive Voice Meets the “In-Group” Effect

**DOI:** 10.3390/bs16030428

**Published:** 2026-03-16

**Authors:** Kaiyin Zhong, Junchen Shang

**Affiliations:** Department of Medical Humanities, School of Humanities, Southeast University, Nanjing 211189, China; 220234531@seu.edu.cn

**Keywords:** beauty premium, in-group favoritism, trust, feedback

## Abstract

Prior research has revealed the isolated effects of voice attractiveness and group identity on trust decisions. This study investigated how males’ voice attractiveness and group identity simultaneously influenced females’ trust decisions as trusters (Experiment 1) and trustees (Experiment 2). Experiment 1 showed that trustees with attractive voices were more likely to receive initial investments, and in-group trustees obtained greater investments when feedback was unknown. However, upon receiving feedback, these effects vanished. Experiment 2 showed that participants expected trusters with attractive voices to have higher investment amounts. Participants reciprocated more to in-group trusters than to out-group trusters. In summary, voice attractiveness and group identity influenced trust decisions independently, and the effects were context-dependent. Moreover, the effects of the “beauty premium” and “in-group favoritism” in trust-based decision-making varied depending on role, feedback, and stage of the decision-making process.

## 1. Introduction

As illustrated by the idiom “a voice is so sweet that it lingers on the eaves for three days without fading”, nowadays, humans’ love for and pursuit of melodious voices remains as steadfast as ever. Voice attractiveness refers to “the degree to which a target individual’s voice elicits a positive, pleasurable emotional experience and motivates others to approach them” (Zheng et al., 2017). Research has shown that voice attractiveness can trigger a “beauty premium” effect, whereby individuals with attractive voices often receive preferential treatment in social decision-making—such as receiving more votes (Tigue et al., 2012), being perceived as more competent leaders (DeGroot et al., 2011), having more sexual partners (Hughes et al., 2004), and being judged as more physically attractive (Feinberg et al., 2005). Moreover, Rezlescu et al. (2015) revealed a positive correlation between voice attractiveness and perceived trustworthiness—meaning that the more attractive a voice sounds, the higher its perceived trustworthiness. These findings demonstrated that the voice attractiveness premium is not a simple effect but rather a complex interaction embedded within social role expectations. Driven by voice attractiveness, the “beauty premium” phenomenon extends its influence far beyond the specific contexts mentioned above, serving as a latent social cognitive bias that pervasively shapes various dimensions of interpersonal judgment. As the cornerstone of human interaction, trust decisions naturally become a core domain where this effect unfolds—exploring how voice attractiveness shapes judgments in this process is essential for understanding its profound social implications.

The trust game paradigm provided a classic framework for quantifying trust decisions (Berg et al., 1995). In the game scenario, the truster first decides whether to invest funds in the trustee, with the investment rate or amount directly reflecting the level of trust. Once the trustee receives an investment, the funds will be multiplied by a factor ranging from 2 to 4. The trustee further decides whether to return part of the funds to the truster. The rate or amount returned corresponds to the trustee’s feedback of trust towards the truster. Shang and Liu (2022a, 2022b) employed a trust game and found that participants invested more in trustees possessing attractive voices. Additionally, Shang and Zhang (2025) discovered that trustees with attractive voices received higher initial investments from participants as trusters. When trustees did not reciprocate, participants still reinvested in trustees with attractive voices. Interestingly, when participants served as trustees, their repayment decisions were unaffected by the trusters’ voice attractiveness. Although some studies have investigated the role of voice attractiveness in trust games, few have examined the boundary conditions that may moderate the beauty premium effect. For example, group identity is a potential moderating variable worthy of investigation.

In daily life, we encounter people from different social groups. Group identity plays a pivotal role in trust decision-making. Group identity refers to an individual’s recognition of their group affiliation, along with the associated values and emotions attached to that identity (Zhang et al., 2020). It directly influences behavior in intergroup interactions, such as the in-group favoritism effect, which manifests specifically in individuals allocating positive attitudes and greater resources to members of their own group (e.g., Bilancini et al., 2020). According to Social Identity Theory (SIT; Tajfel & Turner, 1979), individuals construct their identities through group membership and tend to trust in-group members. For example, Burnham et al. (2001) manipulated the interaction relationship (partner versus opponent) in the trust game and found that participants exhibited significantly higher levels of trust in the partner condition than in the opponent condition. Moreover, Ioannou et al. (2015) found that the initiating party in the trust game exhibited a higher investment rate towards in-group members than towards out-group members. Similarly, Shen et al. (2022) reported that participants invested more in in-group members. Therefore, whether the truster and the trustee share a common group identity may moderate the influence of interpersonal cues (e.g., voice attractiveness) on trust decisions. Prior research has shown that group identity—when signaled through vocal cues—moderates the link between voice and trust. For example, Jiang et al. (2020) found that English Canadian listeners judged statements as more trustworthy when they were spoken in a confident voice by in-group speakers (i.e., those sharing their dialect) compared to out-group speakers (e.g., regionally or foreign-accented). In their work, group membership was defined auditorily, based on accent. However, in many real-world interactions, group identity is often conveyed through visual cues (e.g., race, clothing, or symbols) rather than voice alone. It remains unclear how voice attractiveness and visually cued group identity jointly shape trust decisions—a gap the present study aims to address.

Shang et al. (2025) examined the influence of visual group identity and voice attractiveness on ultimatum decision-making, identifying an independent “in-group favoritism” effect (Tajfel et al., 1971) and a “beauty premium” effect, while there was no interaction between the two factors. Nonetheless, the manipulation of group identity in Shang et al. (2025) was overly simplistic, with participants divided into in-group and out-group without a control group, resulting in low ecological validity of the findings. Moreover, they only examined responders’ decisions towards proposers in the ultimatum game, but it is unclear whether the effect of group identity will change depending on participants’ roles in the trust game. To address these limitations, this study follows the approach of previous researchers (Ahmed, 2007; Schiller et al., 2014; Vermue et al., 2019) by employing an unknown group as the control group to investigate the effects of voice attractiveness and group identity on trust decisions.

In summary, this study aims to examine the joint influence of voice attractiveness and group identity on trust decisions, introducing a multi-stage feedback mechanism to better capture the dynamic decision-making process. The trust game involves two roles: in Experiment 1, participants acted as the truster; in Experiment 2, participants acted as the trustee. Experiment 1 employed the minimal group paradigm (MGP; Otten, 2016) to assign trustees to in-group, out-group, or unknown-group conditions. Participants decided on the investment amount based on the trustees’ (from different groups) attractive/unattractive voices, and then determined whether to continue investing in the future after receiving the trustees’ feedback (gain or loss). Experiment 2 employed MGP to manipulate the group membership of trusters. Participants (acting as trustees) predicted the investment amounts of attractive/unattractive voice trusters (from different groups) and, upon receiving investments, determined whether to reciprocate. To simplify the research design and avoid potential complex interaction effects arising from gender factors, this study recruited only female participants and uniformly employed male voices as auditory stimuli, following a similar research design (Shang et al., 2025).

We propose the following hypotheses.

During the initial decision-making phase, whether serving as a truster or trustee, participants can only make heuristic judgments based on limited surface cues (e.g., voice attractiveness and group identity) before actual interaction with the counterpart, thereby determining investment or predictive behaviors. Consequently, the same cues could elicit similar behavioral effects in both roles. H1: As trusters, participants allocate higher investment amounts to trustees with attractive voices prior to receiving feedback (Shang & Liu, 2022a, 2022b; Shang & Zhang, 2025), and higher investment amounts to in-group trustees (Burnham et al., 2001; Shen et al., 2022; Vermue et al., 2019). H2: When participants act as trustees unaware of the truster’s investment decisions, they predicted higher investment amounts for trusters with attractive voices and in-group trusters compared to other conditions (Shang & Zhang, 2025).

After the initial investment phase, participants have actual communication with their counterparts. Therefore, their subsequent decisions no longer rely solely on surface cues but increasingly draw upon behavioral feedback and interactive experiences from their counterparts, potentially leading to distinct behavioral effects between reciprocation and reinvestment. H3: If acting as trusters receiving gain feedback, participants may demonstrate higher reinvestment amounts for trustees with attractive voices (Shang & Zhang, 2025) and for trustees from the in-group (exploratory hypothesis). However, upon receiving loss feedback, the brain reward circuitry activated by attractive voices may counteract loss aversion (Shang & Liu, 2022a, 2022b). Positive attitudes towards in-group trustees could also attenuate the loss aversion (exploratory hypothesis), potentially neutralizing both voice attractiveness and group identity effects. H4: When participants act as trustees receiving investments, their return decisions are unaffected by voice attractiveness (Shang & Zhang, 2025), but they may return more to in-group trusters (exploratory hypothesis).

## 2. Materials and Methods

### 2.1. Experiment 1: Voice Attractiveness and Group Identity Influence Truster’s Decision-Making

According to G*power 3.1.9.7 estimation (using repeated measures analysis of variance, setting statistical power at 0.95, effect size at 0.25, and measurement number at 4), a minimum of 36 participants is required. A total of 60 female students (*M*_age_ = 20.67 years, *SD* = 1.70) from non-economics and non-psychology majors at Southeast University were recruited. All participants reported no history of mental health issues, had never participated in similar experiments, and had normal hearing and normal or corrected-to-normal vision. This research was approved by the Ethics Committee of the Psychology Research Center at Southeast University (hereinafter referred to as the same). All participants voluntarily took part and signed informed consent. They received appropriate compensation upon completion of the experiment.

The voice stimuli were selected from Ferdenzi et al. (2015), comprising 111 voice samples (61 female voices and 50 male voices). Each voice sample contained three non-meaningful vowels: /i/, /a/, and /ou/. All voice samples were standardized to a duration of 2040 ms and a uniform loudness of 70 dB in mono. Shang and Liu (2022a, 2022b) recruited participants to rate the attractiveness of these voices on a 7-point scale (1 = very unattractive, 7 = very attractive). Based on average voice attractiveness ratings, 12 attractive and 12 unattractive male voices were ultimately selected for the formal experiment (see Table 1 for descriptive statistics). This study divided these voice stimuli into three groups, with 8 voices in each group. A two-factor ANOVA was conducted on the attractiveness ratings of the 24 voices used in the formal experiment. The main effect of voice attractiveness was significant, *F*(1, 56) = 141.49, *p* < 0.001, and *η_p_*^2^ = 0.876. Neither the main effect of group identity nor the interaction between the two factors was significant, *F*s ≤ 0.063, *p*s ≥ 0.805. From the remaining materials, 3 attractive and 3 unattractive male voices were selected as stimuli for the practice phase.

Previous research has found that the fundamental frequency (F0) of the voice was associated with perceived trustworthiness (Belin et al., 2017); therefore, it may serve as a potential confounding variable in the present study. To address this, we used Praat Chinese Localized Version 5.3 software and scripts to compute acoustic parameters of the voice stimuli, followed by independent-samples *t*-tests. The results showed no significant difference in F0 between attractive voices (*M* = 126.12, *SD* = 17.13) and unattractive voices (*M* = 142.73, *SD* = 29.96), *t*(17) = −1.67, *p* = 0.113. These findings suggested that the observed effects in our study were driven by attractiveness itself.

This experiment employed a 2 (voice attractiveness: attractive, unattractive) × 3 (group identity: in-group, out-group, and unknown-group) within-participant design. The dependent variables comprised the initial investment amount and the reinvestment amount (Shang & Zhang, 2024; Wilson & Eckel, 2006). The experiment was adapted from the trust games described by Wilson and Eckel (2006) and Jaeger et al. (2022). Participants acted as trusters throughout the task. The experiment consisted of four sequential phases: a personality test, a pre-experimental voice recording and questionnaire completion, a trust game, and a post-experimental evaluation task.

Prior to arriving at the laboratory, participants were required to complete a questionnaire measuring personality traits (Wu et al., 2015). This questionnaire corresponded to the 40 items of the Eysenck Personality Questionnaire (Eysenck & Eysenck, 1975) and employed a binary response format of “yes/no”. This test is a sham assessment tool. Its results bear no relation to the participants’ actual personality traits. This stage was designed solely to bolster the perceived legitimacy of group categorization, thereby ensuring the subsequent manipulation of group identity is effective. Upon completion of the questionnaire, participants received feedback indicating their personality type as either the red personality or the blue personality, and were subsequently grouped accordingly. Half of the participants were assigned to the red group, while the other half were assigned to the blue group.

Upon arrival at the laboratory, participants first read and signed the voice recording agreement. They were told that they would initially play a truster in this experiment and later play a trustee in follow-up experiments. Next, the experimenter guided participants to record their voices using their mobile phones (nonsense vowels identical to the experimental stimuli). They were requested to send the recorded voice to the experimenter after completing this experiment. Participants also completed the return decision questionnaire as trustees. They were informed that their questionnaire responses, together with their recorded voices, would constitute the trustee experimental materials to be subsequently presented to other participants acting as trusters. This manipulation was designed to further make participants believe that the trustee’s voice and the corresponding investment decision feedback were from real people. In fact, the trustee’s questionnaire and voice recording task were used to enhance the authenticity of the experimental scenario. The experimenter did not actually have the participants send voices in the end.

Participants sat in a laboratory at a distance of approximately 55 cm from the screen, wearing Sennheiser earphones. The experiment was conducted on a ThinkPad T14 laptop (14-inch display, 2440 × 1400 resolution, and 60 Hz refresh rate). Subsequently, participants were informed that the personality test they had completed earlier would be used for group allocation. They were then fitted with wristbands in the color corresponding to their group, while labels of the same color were affixed to the right-hand side of the screen. This approach reinforced participants’ perception of their group identity through visual cues. Participants were informed of the rules of the trust game and that they would receive 120 tokens. The token amount was determined based on the number of trials, ensuring that even if participants selected the maximum investment amount in each trial, tokens were sufficient for investment until the experiment concluded. Participants were told their token balance fluctuated in real time according to their investment decisions and the trustee’s returns in the game. The greater the accumulated token balance, the higher the final compensation received. Participants were told that all trustees were students from local universities and had all completed voice recording and decision-making questionnaires in advance. To establish a baseline comparison group identity (unknown-group), the experimenter explained to participants that some trustees had joined the experiment without completing the personality test due to the urgency of recruitment, thus their personality type remained unknown.

**Figure 1:**
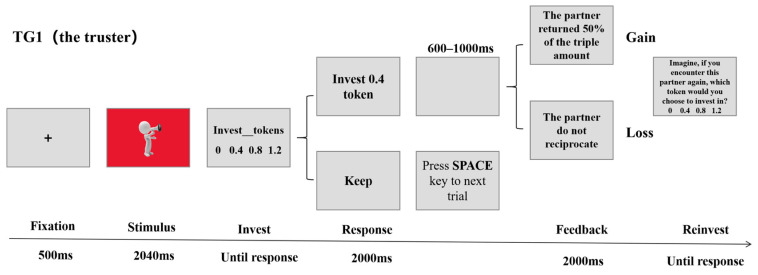
The trust game process under the role of a truster.

At the start of each trial, a fixation point appeared centrally on the screen for 500 ms, followed by the simultaneous presentation of the trustee’s voice and personality type for 2040 ms. Personality types were represented by screen background colors: red personality, blue personality, and green for unknown personality. Accordingly, participants may categorize the trustee as an in-group, out-group, or unknown-group. After the trustee’s information disappeared, the participant selected the amount to invest, with options of 0, 0.4, 0.8, or 1.2 tokens. Next, the investment decision was displayed for 2000 ms. If participants chose to invest, the experimenter would triple the investment amount and give it to the trustee. Subsequently, the trustee’s feedback information (keep all/return half) would be displayed for 2000 ms after a blank screen was shown randomly, ranging from 600 to 1000 ms. Then, the participants imagined how many tokens they were willing to invest if they encountered the trustee again. The next trial began after the decision was made. The procedure is illustrated in Figure 1. The formal experiment consisted of 96 trials, including 48 trials for gain feedback and 48 trials for loss feedback. The experiment was divided into 4 blocks, each containing 24 trials. The voices of the 24 trustees appeared only once in a random order within each block. Participants rested briefly after completing each block. Prior to the formal experiment, participants completed 24 practice trials. The voice stimuli used in the practice trials did not duplicate those employed in the formal experiment.

After the formal experiment, participants rated the attractiveness of voices that appeared in the formal experiment on a 7-point scale (1 = very unattractive, 7 = very attractive).

After the experiment, all participants reported not guessing the true purpose of the experiment and believed in the authenticity of the investment game task, indicating effective manipulation of the experimental situation.

### 2.2. Experiment 2: Voice Attractiveness and Group Identity Influence Trustee’s Decision-Making

As in Experiment 1, G*Power estimated a minimum sample size of 36 participants. Sixty-two female participants (*M*_age_ = 20.77 years, *SD* = 2.02) who were not majoring in psychology or economics were recruited from Southeast University. All participants reported good mental health and possessed normal hearing. They had normal or corrected-to-normal vision. All participants had never previously participated in similar experiments and voluntarily took part in this experiment. They all signed an informed consent form, receiving appropriate compensation upon completion of the experiment. The experimental stimuli were the same as those used in Experiment 1.

This experiment employed a 2 (voice attractiveness: attractive, unattractive) × 3 (group identity: in-group, out-group, and unknown-group) within-participant design. The dependent variables were expected investment amount and reciprocation rate (Shang & Zhang, 2024). The experiment was adapted from the trust games described by Jaeger et al. (2022) and Wilson and Eckel (2006), requiring participants to act as trustees. This experiment consisted of four sequential components: a personality test, pre-experimental voice recording and questionnaire completion, a trust game, and a post-experimental evaluation task. The procedure prior to the formal experiment largely mirrored that of Experiment 1. The key difference lay in the following: to enhance the authenticity of the game, participants were required not only to complete the trustee task but also to complete an investment decision questionnaire as a truster.

**Figure 2:**
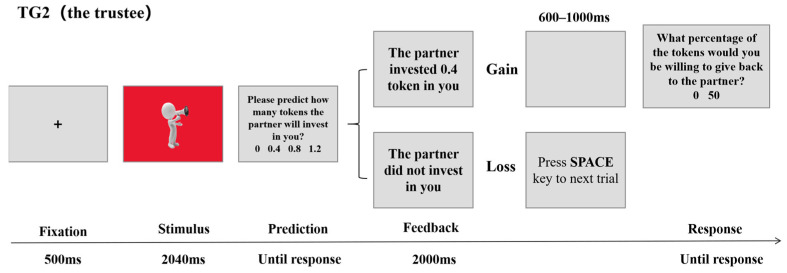
The trust game process under the role of a trustee.

At the start of each trial, a fixation point appeared at the center of the screen for 500 ms. Subsequently, the screen simultaneously displayed the truster’s voice and personality type, which was represented by screen background colors: red personality, blue personality, and green for unknown personality. Accordingly, participants could categorize the truster as an in-group, out-group, or unknown-group member, with the stimulus remaining displayed for 2040 ms. Participants were then required to predict how many tokens the truster would invest in them, with the following options: 0, 0.4, 0.8, or 1.2 tokens. To enhance engagement in the task, participants were told they would receive one token for each correct prediction. After the participants made their prediction, the screen displayed the truster’s actual investment choice for 2000 ms. If the participants received an investment, the amount would be tripled. After a blank screen presented randomly ranging from 600 to 1000 ms, the participants chose to keep all the income or return half of the income to the truster. After making the decision, the next trial began. If the participants did not receive an investment, they would directly enter the next trial. The formal experiment included a total of 96 trials, with 24 trials for each of the truster’s investment amounts (0, 0.4, 0.8, and 1.2 tokens). The experiment was divided into 4 blocks, each containing 24 trials, and 24 trusters’ voices presented in each block were not repeated, with a random order. Prior to the formal experiment, participants completed 24 practice trials. The procedure is illustrated in Figure 2. The participants took a break after completing each block.

The post-experimental evaluation task was the same as that in Experiment 1. After the experiment, all participants reported not knowing the true purpose of the experiment and believed in the authenticity of the investment game, indicating effective experimental manipulation.

## 3. Results

The experimental data was analyzed and processed using IBM SPSS 27.0. A repeated measures ANOVA was performed on each dependent variable with voice attractiveness and group identity as independent variables. Where data failed to satisfy the sphericity test, the Greenhouse–Geisser method was employed for correction. All multiple comparisons were adjusted using the Bonferroni correction method. Experiment 2 was identical to Experiment 1. The descriptive statistics for each condition are presented in Table 2.

### 3.1. Manipulation Check of Stimuli Selection

In Experiment 1, a two-factor ANOVA on post-test scores for voice attractiveness revealed a significant main effect of voice attractiveness. The ratings for attractive voices (*M* = 4.00, *SD* = 0.07) were significantly higher than those for unattractive voices (*M* = 2.88, *SD* = 0.07), *F*(1, 354) = 134.160, *p* < 0.001, and *η*_p_^2^ = 0.275. Neither the main effect of group identity nor the interaction between the two factors was significant, *F*s ≤ 1.198, *p*s ≥ 0.303.

In Experiment 2, the results of a two-factor ANOVA on post-test scores revealed a significant main effect of voice attractiveness. The ratings for attractive voices (*M* = 4.13, *SD* = 0.06) were significantly higher than those for unattractive voices (*M* = 2.92, *SD* = 0.06), *F*(1, 366) = 192.279, *p* < 0.001, and *η*_p_^2^ = 0.344. Neither the main effect of group identity nor the interaction between the two factors was significant, *F*s ≤ 1.894, *p*s ≥ 0.152.

The above results indicate that participants’ perceptions of voice attractiveness in the experiment corresponded with the trend observed in pre-selection ratings, confirming the validity of the stimuli manipulation.

### 3.2. Tg1: Initial Investment Amount

**Figure 3:**
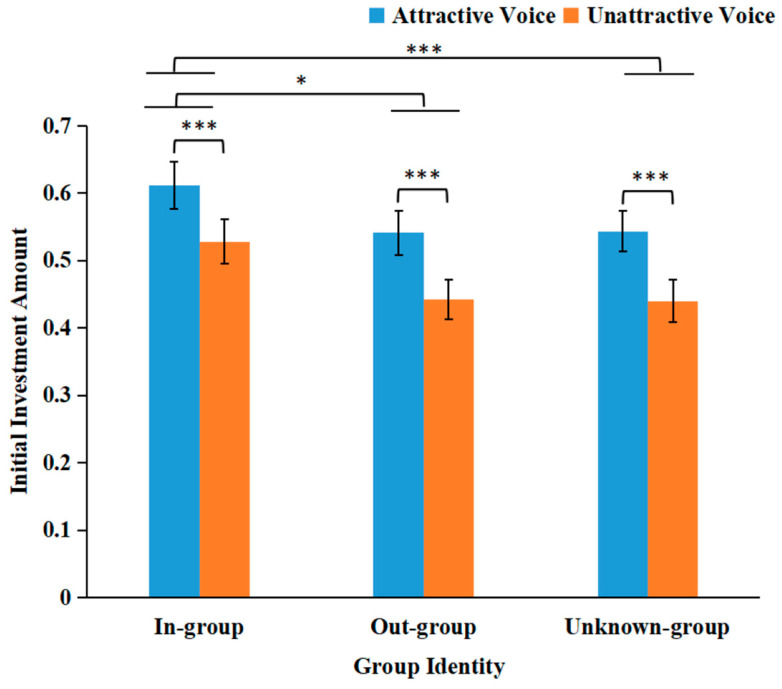
Mean initial investment amounts as a function of voice attractiveness and group identity. *** *p* < 0.001, and * *p* < 0.05.

As Figure 3 illustrates, the main effect of voice attractiveness was significant, as *F*(1, 59) = 41.732, *p* < 0.001, and *η*_p_^2^ = 0.414. Participants gave more investment when trustees had attractive voices (*M* = 0.565, *SD* = 0.029) than when they had unattractive voices (*M* = 0.470, *SD* = 0.027). The main effect of group identity was significant: *F*(2, 96) = 6.508, *p* = 0.004, and *η*_p_^2^ = 0.099. The investment amount for the in-group (*M* = 0.570, *SD* = 0.033) was significantly higher than that for the out-group (*M* = 0.491, *SD* = 0.030), *p* = 0.012, and significantly higher than that for the unknown-group (*M* = 0.492, *SD* = 0.029), *p* < 0.001. The interaction between the two factors was not significant: *F*(2, 118) = 0.433, and *p* = 0.649, *η*_p_^2^ = 0.007.

### 3.3. Tg1: Reinvestment Amount

Separate analyses were conducted on the reinvestment amounts for gain and loss feedback trials. Under the gain feedback condition, no main effects and interactions were significant: *F*s ≤ 2.696, *p*s ≥ 0.083. Under the loss feedback condition, no main effects and interactions were significant: *F*s ≤ 1.598, *p*s ≥ 0.209.

### 3.4. Tg2: Expected Investment Amount

**Figure 4:**
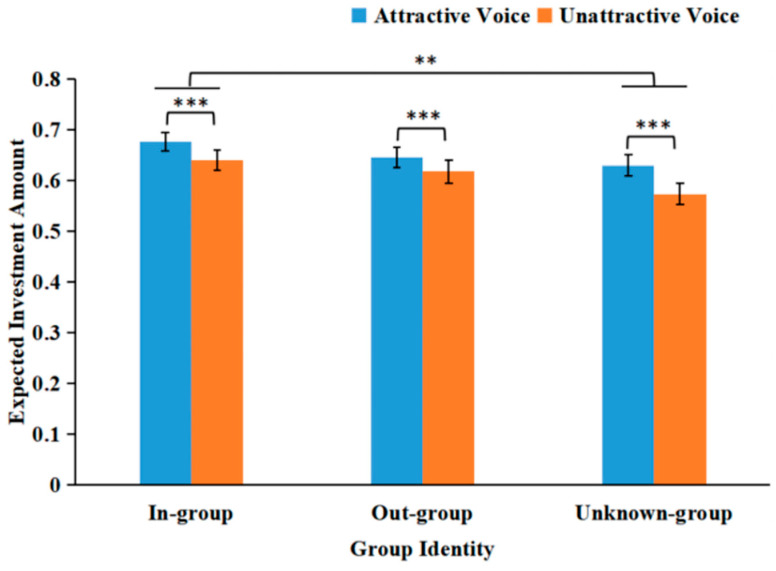
Mean expected investment amounts as a function of voice attractiveness and group identity. *** *p* < 0.001, and ** *p* < 0.01.

We observed a beauty premium (see Figure 4). The main effect of voice attractiveness was significant: *F*(1, 61) = 19.345, *p* < 0.001, and *η*_p_^2^ = 0.242. Participants predicted that trusters with attractive voices (*M* = 0.651, *SD* = 0.015) gave more investment than trusters with unattractive voices (*M* = 0.611, *SD* = 0.016). The main effect of group identity was significant: *F*(2, 122) = 4.194, *p* = 0.017, and *η*_p_^2^ = 0.064. Participants predicted that in-group trusters (*M* = 0.659, *SD* = 0.017) gave more investment than unknown-group trusters (*M* = 0.602, *SD* = 0.019), *p* = 0.002. However, the differences between the in-group and out-group, and between the out-group and unknown-group, were not significant: *F*s ≤ 5.089, *p*s ≥ 0.14. The interaction between voice attractiveness and group identity was not significant: *F*(2, 122) = 0.761, *p* = 0.470, and *η*_p_^2^ = 0.012.

### 3.5. Tg2: Reciprocation Rate

For trials in which participants received investments, their return decisions were incorporated into the analysis.

**Figure 5:**
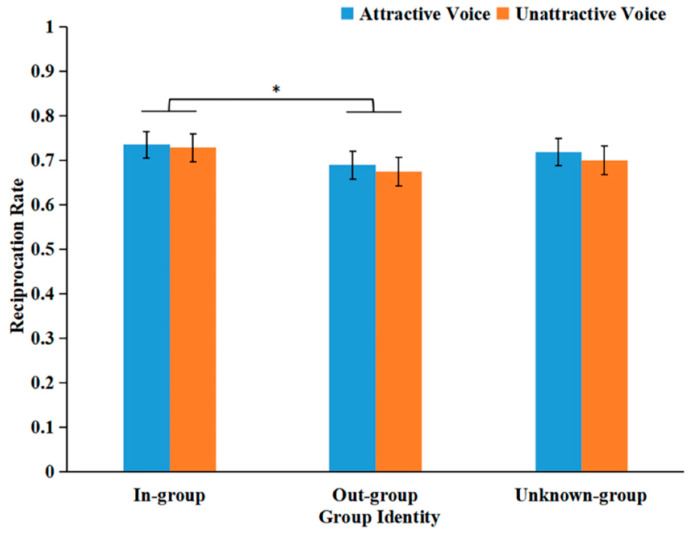
Mean reciprocation rates as a function of voice attractiveness and group identity. * *p* < 0.05.

The main effect of voice attractiveness was not significant: *F*(1, 61) = 3.124, *p* = 0.082, and *η*_p_^2^ = 0.049. We observed in-group favoritism. The main effect of group identity was significant (see Figure 5): *F*(2, 108) = 4.313, *p* = 0.019, and *η*_p_^2^ = 0.066. Participants were more likely to reciprocate in-group trusters (*M* = 0.732, *SD* = 0.029) rather than out-group trusters (*M* = 0.682, *SD* = 0.031), *p* = 0.014. However, the differences between the in-group and unknown-group, and between the out-group and unknown-group, were not significant: *F*s ≤ 3.135, *p*s ≥ 0.088. The interaction between the two factors was not significant: *F*(2, 122) = 0.122, *p* = 0.886, and *η*_p_^2^ = 0.002.

## 4. Discussion

This study examined the influence of voice attractiveness and group identity on trust decisions through two experiments. Experiment 1 uncovered a beauty premium effect and an in-group favoritism effect in initial investment amounts. Experiment 2 further revealed the beauty premium in the expected investment amounts through the transformation of participants’ roles. Additionally, the in-group favoritism effect emerged in the reciprocation behavior. Furthermore, consistent with Shang et al. (2025), who observed isolated effects of voice attractiveness and group identity, there were no interactions between voice attractiveness and group identity in any decision-making stage in this study.

Our study found that voice attractiveness induced the “beauty premium” effect only during the initial phase of the decision-making process and was unaffected by the role of participants in the game. Specifically, Experiment 1 found that participants acting as trusters tended to allocate more tokens to trustees with attractive voices (Shang & Zhang, 2025; Shang & Liu, 2022a, 2022b), partially supporting Hypothesis 1. Experiment 2 revealed that participants acting as trustees anticipated that trusters with attractive voices would invest more tokens in them, supporting Hypothesis 2 and aligning with the findings of Shang and Zhang (2025). One possible reason was that in the early stages of decision-making, there was not enough information for complex cost–benefit calculations, and voice attractiveness served as a cue to primarily influence decisions by activating the “attractiveness–trustworthiness” stereotype. Specifically, when participants heard attractive voices, they automatically assumed the counterparts had positive traits such as “high credibility, high willingness to cooperate, and high potential for reward” (Li & Chen, 2020).

However, voice attractiveness only affected initial one-shot decisions rather than the long-term interactions during the later stages of decision-making. Specifically, no discernible differences attributable to voice attractiveness were observed either in the reinvestment phase of Experiment 1 or the return phase of Experiment 2. This is inconsistent with Hypotheses 3 and 4, reflecting the fragility and instability of the voice attractiveness effect within complex decision-making contexts. One possible explanation was that in the reinvestment stage of the trust game, participants decided whether they would cooperate with their counterparts in the future, and they already knew the outcome of their initial decision. The reinvestment decision involved explicit calculation of rewards and rational weighing of risk costs, which may hinder the earlier intuitive responses driven by stereotypes of voices, as attractiveness was an implicit cue. For example, Torre et al. (2015) employed a trust game in which participants acted as trusters and heard sentences spoken by virtual counterparts with different accents before deciding how much to invest in them. Upon learning of the counterpart’s reciprocation behavior, participants adjusted subsequent investments based on whether the counterpart demonstrated generosity or selfishness. Shang and Zhang (2025) found that, regardless of whether participants received gain or loss feedback, they maintained higher levels of reinvestment towards counterparts with attractive voices, which differs from the findings of this study. This discrepancy may stem from multiple factors: Firstly, Shang and Zhang (2025) incorporated a social interest variable into their experiment and presented only two options during the reinvestment phase, focusing their analysis on the reinvestment rate. In contrast, this study offered four reinvestment options and analyzed outcomes based on reinvestment amounts. These methodological differences may account for the divergent results. Secondly, their experiments employed authentic sentences as auditory stimuli, whereas this study utilized non-meaningful vowels. The latter inherently elicits a weaker and less sustained attractiveness effect. In addition, Experiment 2 aligned with the null effect of voice attractiveness on the reciprocation rate reported by Shang and Zhang (2025). When participants had the authority to divide money, they did not need the consideration of an implicit cue, such as voice attractiveness.

Consistent with the phenomenon of “in-group favoritism” within the SIT framework (Tajfel et al., 1971), this study indicated that participants invested significantly more in in-group trustees than in out-group or unknown-group trustees in the initial investment stage. The investment amounts reflected the depth of trust: when dealing with in-groups, a heightened sense of group belonging fostered trust, thereby widening the investment gap with other groups. This partially supported Hypothesis 1, namely that in-group identity can elicit stronger trust responses (Tang & Gong, 2024). Less investment in out-group trustees was attributed to participants’ negative expectations regarding their cooperative intentions (Judd et al., 2005). For the unknown-group, the uncertainty about their identity further induced risk-averse tendencies among participants. However, no significant difference in investment was found between the out-group and unknown-group. This finding was inconsistent with conclusions from baseline control studies of similar design (Ahmed, 2007; Schiller et al., 2014; Vermue et al., 2019). We speculated that these discrepancies may be attributable to the experimental tasks employed. For instance, Ahmed (2007) observed that in both the prisoner’s dilemma game and the stag hunt game, participants exhibited the highest propensity for cooperation towards their in-group, followed by an intermediate level towards the unknown-group, and the lowest towards the out-group. Similarly, Schiller et al. (2014) observed in a third-party punishment game that participants imposed the most severe penalties on the out-group, followed by the unknown-group, and the mildest penalties for the in-group. However, although Vermue et al. (2019) employed a trust game, in this study, participants acted as trusters, making decisions after viewing party information (without voice manipulation). The results revealed significant between-group differences during the initial stages of trust formation (the first three rounds). In this study, drawing on cognitive load theory, we propose a speculative interpretation of our findings: when reliable cues are lacking, cautious participants may struggle to effectively distinguish risk levels posed by out-group members versus unknown-group members under limited cognitive resources. This ambiguity may prompt them to adopt a cognitive simplification strategy, categorizing both out-group and unknown-group members into a “high-risk” category to reduce cognitive load during decision-making.

However, no group effect was observed in the amount of reinvestment. On one hand, upon knowing the feedback, the decision-making focus shifted from identity-based preconceptions to verification based on actual interaction outcomes. At this stage, whether an individual reciprocated became the core basis for gauging the trustee’s willingness to cooperate and credibility. Consequently, the influence of group identity may diminish. On the other hand, the quality of contact between groups may also be a key reason for the absence of group effects during reinvestment. Herrera and Kydd (2022) revealed the prerequisites for establishing trust between groups. If interactions between participants and members of different groups during the study remained at the superficial level of “one-off returns,” without forming sustained information exchange or emotional bonds, group identity may struggle to translate into a stable foundation for trust. Conversely, the essence of reinvestment decisions lies in a secondary assessment of “future collaborative benefits”—a dimension unrelated to the remuneration used in this experiment. As a result, participants may abandon reliance on group identity.

Furthermore, the persistence of group identity effects differs when participants act as trusters and trustees: the group effect was confined to the initial investment phase in Experiment 1, as trusters’ initial decisions prioritized personal gains and losses alongside outcome uncertainty. In Experiment 2, however, the effect became markedly more complex and dynamic throughout the entire decision-making process. This occurred because the authority over the money division changed, and the nature of risk differed between the early and late stages. As trustees, participants were more likely to expect in-group trusters to invest in them relative to the unknown-group trusters, partially supporting Hypothesis 2. However, no significant differences were observed between the out-group and the other two groups. This pattern of results may be explained by integrating the SIT and the uncertainty avoidance mechanism. In-group trusters activated individuals’ positive expectations regarding “in-group cooperative norms”, leading them to perceive the in-group as a credible extended exchange system (Kiyonari, 2002). For the unknown-group, participants faced uncertainty about whether the truster was in the in-group, resulting in decision-making difficulties. Consequently, adhering to the principle of risk aversion (Nie et al., 2024), participants tended to anticipate less investment amount from the unknown-group compared to the in-group. Moreover, for the out-group, participants formed automatic categorizations of group identity in decision-making. Nonetheless, as trustees, participants had no authority over the money division, and thus, intergroup competition was relatively weak. The out-group trusters may not be perceived as a clear threat. Also, from the perspective of the rational economic agent, individuals must first invest to potentially achieve higher returns. Therefore, out-group trusters may also invest for profit motivation, and participants could not ascertain whether their investment willingness was genuinely lower than the other two groups. Consequently, participants adopted more conservative strategies toward out-group members than toward unknown-group members.

During the reciprocation phase, a different group effect emerged. Participants exhibited significantly higher reciprocation rates towards the in-group than the out-group, partly supporting Hypothesis 4. According to SIT (Tajfel et al., 1971), upon receiving investments, participants evaluated their in-group more positively and were therefore more inclined to reciprocate in-group trusters, thereby reinforcing group identity and their own sense of belonging (Zhang et al., 2022). However, no significant differences were observed in return decisions between the unknown-group and the in-group or out-group. This may be because, upon learning the investment amount, participants adopted differentiated strategies towards clearly defined groups (in-group and out-group): maintaining a persistent preference for the in-group while simultaneously devaluing the out-group. For the unknown-group, there was still no clear information about group affiliation. While their investment provided positive clues, there were equal trials in which they did not invest. Consequently, this resulted in insufficiently pronounced differences in reciprocation rates compared with in-group and out-group trusters.

The current study has several limitations. Firstly, it recruited only female participants and used only male voices—a design choice made primarily to test a “simplified yet controlled” theoretical model under constraints of resources and sample size. Moreover, women generally demonstrate superior voice recognition abilities compared to men (Fecher & Johnson, 2021). Although this choice helps establish preliminary evidence for the existence of an interaction effect between voice attractiveness and group identity at a baseline level, it does strictly limit the generalizability of the findings to the specific gender pairing context in which female participants assess male voices. Extensive literature has demonstrated that intrasexual competition and intersexual selection lead to differential perceptions of male and female voices across contexts. For instance, preferences for pitch height exhibit systematic variations depending on the speaker’s gender (Apicella & Feinberg, 2009; Re et al., 2012). Consequently, the conclusions of this study are inherently limited, and certain questions remain insufficiently examined. Future research should adopt a fully factorial design of 2 (participant gender) × 2 (speaker gender) × 2 (voice attractiveness) × 2 (group identity), supplemented with an adequate sample size, to systematically examine the moderating role of gender pairing in the effects of voice attractiveness and group identity. Secondly, the present study used nonsense vowels as voice stimuli. Although this approach helps control for semantic interference and allows precise manipulation of acoustic features, it comes at the cost of ecological validity. Given that women are more sensitive to prosodic and emotional cues and tend to rely more heavily on social information to mitigate decision-making risks (Croson & Gneezy, 2009), future research should progressively incorporate standardized semantically neutral sentences and further manipulate the emotional valence of semantic content to systematically examine the boundary conditions of voice attractiveness effects across different speech contexts. Thirdly, the interpretation of our findings regarding intergroup differences in the trust game remains somewhat speculative. This is because the current study design did not directly measure participants’ cognitive load levels, nor did it systematically examine the judgment strategies they actually employed during trust decisions. Therefore, this interpretation requires further validation. Future research could employ process-tracing techniques (e.g., response time analysis, cognitive load manipulation) to conduct more direct empirical tests of this underlying psychological mechanism.

## 5. Conclusions

Regardless of whether participants acted as trusters or trustees in the trust game, they favored collaborators with attractive voices and in-group collaborators in the initial decision phase. Neither voice attractiveness nor group identity affected the reinvestment decision. Participants predicted that in-group trusters would invest more than unknown-group trusters, whereas their reciprocation for in-group trusters was higher than that for out-group trusters.

## Figures and Tables

**Table 1:** Means and standard deviations of attractiveness ratings for 24 voices in the formal experiment.

	In-Group	Out-Group	Unknown-Group
Attractive voices	5.07 (0.49)	5.05 (0.33)	4.94 (0.26)
Unattractive voices	2.59 (0.71)	2.87 (0.37)	2.85 (0.54)

**Table 2:** Means and standard deviations of initial investment amount, reinvestment amount under different conditions in TG1, expected investment amount, and reciprocation rate under different conditions in TG2.

	Attractive Voice-In-Group	Attractive Voice-Out-Group	Attractive Voice-Unknown-Group	Unattractive Voice-In-Group	Unattractive Voice-Out-Group	Unattractive Voice-Unknown-Group
Initial investment amount	0.61 (0.27)	0.54 (0.25)	0.54 (0.23)	0.53 (0.26)	0.44 (0.23)	0.44 (0.24)
Reinvestment amount (gain)	0.93 (0.23)	0.87 (0.25)	0.93 (0.23)	0.91 (0.24)	0.88 (0.24)	0.89 (0.24)
Reinvestment amount (loss)	0.09 (0.13)	0.08 (0.18)	0.06 (0.12)	0.09 (0.16)	0.07 (0.14)	0.08 (0.13)
Expected investment amount	0.68 (0.15)	0.65 (0.16)	0.63 (0.16)	0.64 (0.15)	0.62 (0.18)	0.57 (0.16)
Reciprocation rate	0.74 (0.23)	0.69 (0.25)	0.72 (0.24)	0.73 (0.25)	0.67 (0.26)	0.70 (0.25)

## Data Availability

The data supporting the conclusions of this article are available on OSF (https://osf.io/69ebj/) (accessed on 27 November 2025).
